# Transforming Growth Factor β1 Increases p27 Levels via Synthesis and Degradation Mechanisms in the Hyperproliferative Gastric Epithelium in Rats

**DOI:** 10.1371/journal.pone.0101965

**Published:** 2014-07-07

**Authors:** Ana P. Z. P. Fiore, Luciana H. Osaki, Patricia Gama

**Affiliations:** Department of Cell and Developmental Biology, Institute of Biomedical Sciences, University of Sao Paulo, São Paulo, SP Brazil; National Institute of Agronomic Research, France

## Abstract

Throughout postnatal development, the gastric epithelium expresses Transforming Growth Factor beta1 (TGFβ1), but it is also exposed to luminal peptides that are part of milk. During suckling period, fasting promotes the withdrawal of milk-born molecules while it stimulates gastric epithelial cell proliferation. Such response can be reversed by exogenous TGFβ1, as it directly affects cell cycle through the regulation of p27 levels. We used fasting condition to induce the hyperproliferation of gastric epithelial cells in 14-day-old Wistar rats, and evaluated the effects of TGFβ1 gavage on p27 expression, phosphorylation at threonine 187 (phospho-p27^Thr187^) and degradation. p27 protein level was reduced during fasting when compared to suckling counterparts, while phospho-p27^Thr187^/p27 ratio was increased. TGFβ1 gavage reversed this response, which was confirmed through immunostaining. By using a neutralizing antibody against TGFβ1, we found that it restored the p27 and phosphorylation levels detected during fasting, indicating the specific role of the growth factor. We noted that neither fasting nor TGFβ1 changed p27 expression, but after cycloheximide administration, we observed that protein synthesis was influenced by TGFβ1. Next, we evaluated the capacity of the gastric mucosa to degrade p27 and we recorded a higher concentration of the remaining protein in pups treated with TGFβ1, suggesting augmented stability under this condition. Thus, we showed for the first time that luminal TGFβ1 increased p27 levels in the rat gastric mucosa by up- regulating translation and reducing protein degradation. We concluded that such mechanisms might be used by rapidly proliferating cells to respond to milk-born TGFβ1 and food restriction.

## Introduction

Transforming growth factor β (TGFβ) is part of super family of peptides [1] that function in different tissue contexts to control development, growth and tumorigenesis [2]. In the gastrointestinal tract, TGFβ isoforms regulate epithelial renewal through effects on cell proliferation [3]–[7], differentiation [8], [9], epithelial mesenchymal transition [10], [11], migration [12], and apoptosis [13]. TGFβ has been detected in human and murine milk [14], [15] and after suckling and oral administration, signaling is activated in gastric epithelial cells through receptors (TβRI and TβRII) and Smads central cascade [6], [15]–[17].

Previous studies showed that the components of TGFβ pathway can be altered in gastric tumorigenesis and they act differently depending on cancer stage, in a way that during early events, TGFβ regulates proliferation, whereas lately, it induces invasion and metastasis [7], [11], [18], [19]. TGFβ is also among the many molecules that take part of the complex ontogenesis of gastric mucosa [8], [20]–[22], and we demonstrated that soon after the gavage of suckling rats with TGFβ1, R-Smads are rapidly phosphorylated and increase the concentration of p27, which leads to cell proliferation inhibition in the stomach [6].

p27 belongs to the Cip/Kip family of peptides (cyclin-dependent kinase inhibitors, CKI) that regulate cell cycle progression. Either the loss of p27 or its low concentration increase turnover rates, which are associated with poor prognosis for tumours, including the gastric cancers [23]–[25]. p27 levels oscillate during cell cycle, and this variation is coordinated mainly by post- translational mechanisms [26]. Accordingly, phosphorylation of specific aminoacids and cellular localization determine the role of p27 on proliferation, cell organization, phenotype and migration. The phosphorylation at threonine 187 (Thr^187^) by CyclinE/Cdk2 complex is a first step for proteasomal degradation [26]–[28]. Nuclear phospho-p27^Thr187^ is recognized by S-phase-kinase-associated protein 2 (Skp2) from SCF complex to be ubiquitylated and degraded [29]–[33]. In epithelial cells, TGFβ signaling targets p27 for stability [6], [34]–[36], and such effect is mediated by TGFβ induction of Skp2 degradation [37].

Because milk-born TGFβ as well as the isoforms expressed by the gastric mucosa are directly involved in the control of epithelium proliferation both during development and tumorigenesis, and p27 plays a central role in cell cycle control, we currently aimed to evaluate the molecular mechanisms involved in the regulation of gastric p27 levels by TGFβ. More specifically, we used fasting condition to induce the hyperproliferation of epithelial cells [6], [38], [39], and evaluated the effects of TGFβ1 gavage on p27 expression, synthesis, phosphorylation at threonine 187 and degradation in the rat gastric mucosa. We found that luminal TGFβ1 increased p27 levels by up- regulating translation and reducing protein degradation, and we suggest that such mechanisms could be used by gastric epithelial cells to respond to milk-born TGFβ1 and food restriction. Therefore, we consider that our results add relevant data to studies on nutrition and development, and as importantly, to others that focus on gastric cancer therapy.

## Materials and Methods

### Animals

Wistar rats from the Department of Cell and Developmental Biology Animal Colony (ICB USP) were used. This study was approved by the Ethical Committee in Animal Use (CEUA protocol 8014/2011) from the Institute of Biomedical Sciences at the University of Sao Paulo. The anesthesia was conducted with a 1∶1 mixture of xilazine and ketamine chloridrates (0.5 ml/100 g body weight, Vetbrands, São Paulo, Brazil), and pups were euthanized with the excess of these drugs. Animals were kept at controlled conditions of temperature, humidity and light/dark cycle (12∶12 h). On the 3^rd^ postnatal day, litters were reduced to 8–9 pups, which were kept with their dams until day 14, when half of the group was separated into aluminium cages to be fasted [6], [40]. Water was offered ***ad libitum***. Different litters were used simultaneously to allow the random assignment to experimental groups. Pups were initially fasted for 90 min [6] and treated as described below, while control fed groups remained with dams.

The TGFβ1 (Peprotech, Rocky Hill, USA) was reconstituted in 4 mM HCl containing 0.1% bovine serum albumin (BSA) according to manufacturer's directions and it was further diluted in Phosphate Buffer Saline (PBS) containing 0.2 mg/ml BSA immediately before use. TGFβ1 solution or vehicle was administered by gavage in a single dose 90 min after the onset of fasting (1.5 ng/g body weight), and such procedure was performed according to previous studies [6], [41]. Gastric samples were collected immediately (0 h) or after 2 and 14 h of TGFβ1 or PBS treatment.

The specific effects of TGFβ1 were also tested through its inhibition by using a monoclonal antibody directed to this isoform (α-TGFβ1, R&D, Minneapolis, USA), which was administrated (30 ng/g body weight) [42] together with the peptide. Gastric samples were collected after 2 h of PBS, TGFβ1, α-TGFβ1 and TGFβ1 combined to α-TGFβ1 treatment.

In order to evaluate if TGFβ1 affected protein synthesis, pups were injected with cycloheximide (Biomol, Plymouth, USA) (i.p. injections to a total of 0.3 µg/g body weight) [43] using a first shot 60 min after fasting onset and a second shot immediately after PBS and TGFβ1 gavage. Samples from the gastric mucosa were collected after 2 h of treatment.

The stomach was excised and opened along the lesser curvature under anaesthesia with a 1∶1 mixture of xilazine and ketamine chloridrates (0.5 ml/100 g body weight, Vetbrands, São Paulo, Brazil), and pups were euthanized with the excess of these drugs. The gastric wall was stretched with the mucosal side up and flushed with a 0.9% saline solution for the specific collections described below.

### RT-PCR

For the total RNA extraction, the gastric mucosa was scraped after 2 h of PBS or TGFβ1 gavage and rapidly frozen in liquid nitrogen. Total RNA was extracted using TRIzol reagent (Invitrogen, Carlsbad, USA), according to manufacturer's directions. The cDNA was generated using 1 µg of RNA in the presence of 2.0 µl of Oligo dT (50 mM), 1.0 µl of dNTP mix (10 mM), 1.0 µl of DTT (100 mM), 2.0 µl of reverse transcription buffer (×10), and 1.0 µl of SuperScript III reverse transcriptase (200 U/µl) (all reagents from Invitrogen) to a total volume of 20 µl. The RT-PCR was performed as follows: incubation at 65°C (5 min) for Oligo dT annealing with the RNA, Superscript III reaction at 50°C (50 min) and inactivation of the enzyme at 85°C (5 min).

For the semi-quantitative PCR, 100 ng of each cDNA, 0.4 µl of dNTP mix (1 mM), 0.6 µl of MgCl_2_ (50 mM), 2.0 µl of polymerase buffer (10×) and 0.2 µl of Taq DNA polymerase (5 U/µl) (all reagents from Invitrogen) were incubated with 0.5 µl of each primer for *p27* (10 µM FW: ACG CCA GAC GTA AAC AGC TC, RV: TCC AAT GCT TTT AGA GGC AGA for 30 cycles). As endogenous control, we used 0.5 µl of *β-actin* primers (10 µM, FW: CTG GGT ATG GAA TCC TGT GG, RV: AGG AGC CAG GGC AGT AAT CT for 23 cycles). The PCR was conducted as: denaturation (30 sec at 94°C), annealing (30 sec at 55°C) and extension (45 sec at 72°C) in thermocycler (Eppendorf, Hamburg, Germany).

### Immunohistochemistry

For immunohistochemistry, stomachs were fixed in 10% formaldehyde, embedded in paraffin and non-serial 6 µm tissue sections were placed on positively charged slides (Fisherbrand, Kennesaw, USA). Sections were cleared of paraffin, rehydrated and peroxidase activity and nonspecific binding were blocked with 0.15% H_2_O_2_ (Sigma, Saint Louis, USA) in methanol and 5% rabbit serum (Jackson Laboratories, West Grove, USA), respectively. Antigen retrieval was performed with citric acid (pH 6.0) in microwave (5 min at high power followed by 3×3 min at medium power), followed by incubation with polyclonal anti-phospho-p27^Thr187^ (2.5 µg/ml, overnight at 4°C, Invitrogen). After the addition of biotinylated-goat anti-rabbit secondary antibody (Jackson Laboratories), the peroxidase activity was developed using 0.05% 3,3′-diaminobenzidine (DAB) (DAKO, Carpinteria, USA) in 50 mM Tris containing 3% H_2_O_2_ and slides were counterstained with Mayer's hematoxylin. Negative controls were incubated with normal serum to replace the primary antibody.

In order to estimate the epithelial population immunolabeled for phospho-p27^Thr187^, we identified the first parietal cell to determine the limit between foveolae and the gastric gland, and then tracked the epithelial cells along the entire gland. We counted the nuclei immunostained or not in a total of 1,200 cells per animal. The labeling index (LI) was obtained as the number of phospho-p27^Thr187^ positive cells/total cells ×100. Sections were observed under light microscope at ×800 (Nikon, Tokyo, Japan) and images were captured in light microscope at ×200 (Olympus, Montreal, Canada) using Image ProPlus v.5.2 (Media Cybernetics, Bethesda, USA).

### Protein extraction

For protein extraction, the gastric mucosa was scraped and stored in 10 mM PMSF (Merck, Darmstadt, Germany) in 20 mM Tris buffered saline (TBS) at −80°C. Samples were homogenized in lysis buffer (20 mM Tris HCl pH 8.0, 150 mM NaCl, 1 mM EDTA, 1% NP40 and 10% glycerol) [44] containing a cocktail of protease and phosphatase inhibitors (1 mM PMSF, 1 mM benzamidin, 1 mM leupeptin; 1 mM aprotinin and 1 mM Na_3_VO_4_, Sigma). Homogenates were centrifuged at 12,000 ***g*** (5 min at 4°C), the supernatant was collected and whole protein concentration measured by Bradford method [45].

### p27 degradation assay

In order to determine p27 degradation rate during fasting and TGFβ1 treatment, pups were fasted for 90 min and gavaged with PBS or TGFβ1 as described above. After 2 h of treatment the samples were collected by scraping and frozen in liquid nitrogen. The assay was performed as previously described [34], in order to test how long the gastric mucosa extracts would take to degrade recombinant p27 under each experimental condition. Briefly, each sample was re-suspended in a buffer containing 50 mM Tris (pH 8.3), 5 mM MgCl_2_, 1 mM DTT and protease inhibitors (1 mM aprotinin, 1 mM PMSF, e 1 mM leupeptin). After the complete homogenization, lysates were centrifuged at 10,000 g for 10 min. Protein concentration was determined by Bradford method and then the extracts (200 µg) were mixed with 60 µl of degradation buffer (the same above containing 2 mM ATP) in the presence of 1 µg of recombinant p27 (AbCam, Cambridge, USA) (Pagano et al., 1995). After different intervals (1 min, 30 min, 3 h, 6 h, and 20 h), aliquots of 10 µl (approximately 30 µg) were re-suspended in 20 µl of sample buffer (0.5M Tris, 10% SDS, 1% Bromophenol Blue, 0.1% β- mercaptoethanol) to stop the reaction, and then each vial was frozen in liquid nitrogen.

### Western blotting

Thirty µg of protein extract were boiled in the sample buffer above, fractionated by 12% SDS-PAGE and then transferred to nitrocellulose membrane (Hybond, GE Healthcare, Buckinghamshire, UK). Proteins were detected by 0.5% Ponceau Red solution, the membrane was washed in 20 mM TBS (pH 7.4) and blocked in TBS containing 0.1% Tween 20 and 5% non-fat dry milk or 1% BSA. We incubated the membranes with monoclonal antibodies against p27 (0.3 µg/ml), Cdh1 (0.2 µg/ml, Calbiochem, Billerica, USA), and polyclonal antibodies against phospho-p27^Thr187^ (0.2 µg/ml, AbCam), cyclin E, Cdk2, and Skp2 (0.2 µg/ml, Santa Cruz Biotechnology). Monoclonal antibody for β-actin (0.01 µg/ml, Sigma) was used as an internal control. Bands were detected using ECL system (GE Healthcare), and the integrated optical densities (arbitrary units) were determined by Image J (1.37v Software, NIH Public Domain, USA) for the different proteins, which were compared to β-actin before fold-change calculation in each experiment.

### Statistical analyses

Results were analyzed using ANOVA followed by Tukey's test to evaluate the effects of TGFβ1 treatment. Student's t test was used to compare controls and TGFβ1- treated groups at each interval during degradation assay. Significance level was set at *P*<0.05.

## Results

### Fasting and TGFβ1 effects on p27 levels and phosphorylation at threonine 187

We have previously shown that gavage with TGFβ1 increases p27 levels and inhibits gastric cell proliferation induced by fasting condition during postnatal development [6], [38]–[40]. Currently, we investigated whether this effect is dependent on the regulation of p27 levels at synthesis and degradation steps.

**Figure 1:**
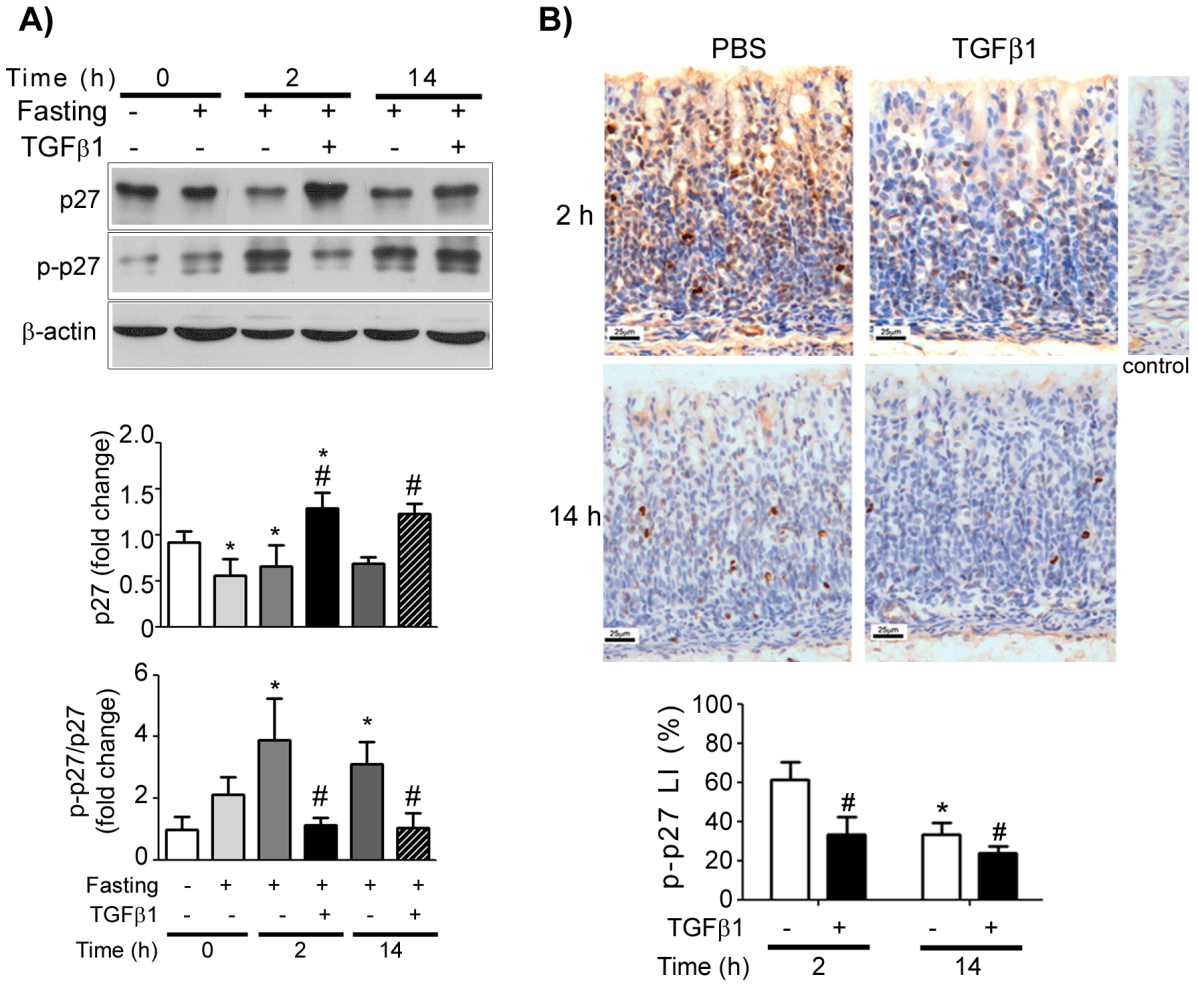
TGFβ1 decreases p27 phosphorylation at Thr187 in the gastric mucosa of 14-day-old rats during fasting. (A) Representative immunoblotting for p27 and phospho-p27^Thr187^ in samples scraped from the gastric mucosa of suckling and fasted pups after 0, 2 and 14 h of PBS and TGFβ1 gavage (1.5 ng/g body weight). β-actin was used as loading control. Each band represents one animal and fold changes are shown as means ± SD for p27 levels and for the ratio of p-p27/p27. (*n*) = 3–4 pups/group. * *P*<0.05 when compared to the suckling group, # *P*<0.05 vs. respective PBS-treated group. Analyses were conducted in blots ran in triplicates. (B) Immunodetection of phospho-p27^Thr187^ in the gastric mucosa of fasted pups after 2 and 14 h of PBS and TGFβ1 gavage. Sections were counterstained with Mayer's Hematoxylin. The labeling index (LI) (%) was obtained as the number of phospho-p27^Thr187^ positive cells/total cells ×100. (*n*) = 4–5 pups/group. Bars represent means ± SD. * *P*<0.05 *vs* PBS control at 2 h; ^#^ *P*<0.05 *vs.* respective PBS-treated group.

We first compared the total concentration and the phosphorylation of p27 at threonine 187. We observed that during food restriction, total p27 levels were reduced when compared to the suckling (control) group (*P*<0.05) (Fig. 1A), and this effect was continuous, as after 14 h of treatment p27 remained low. TGFβ1 administration reversed such response, in a way that 2 h after gavage, we detected a concentration of p27 higher than its corresponding control (*P*<0.05) and also increased when compared to the suckling group (*P*<0.05) that received milk-born TGFβ1. After 14 h, we observed that TGFβ1 effect was maintained, as p27 level was still elevated when the results obtained for pups treated with PBS were considered (*P*<0.05) (Fig. 1A).

By checking phospho-p27^Thr187^ concentration at the intervals used for total p27 analyses, we detected that fasting increased phosphorylation, which reached a peak after 2 h of treatment (Fig. 1A). TGFβ1 gavage reversed this effect and reduced phospho-p27^Thr187^ level (Fig. 1A). In order to compare the rate of phosphorylation, we analysed phospho-p27^Thr187^/p27 ratio, and found that whereas fasting increased it after 2 h (*P*<0.05), TGF β1 reduced the ratio after 2 and 14 h of treatment when the respective counterparts were compared (*P*<0.05) (Fig. 1A).

Through morphological study, we observed that the nuclei immunostained for phospho-p27^Thr187^ were scattered along the gastric gland (Fig. 1B). The labelling index (LI) decreased significantly after TGFβ1 gavage (2 h) when compared to the PBS- correspondent group (*P*<0.05) (Fig. 1B). After 14 h of treatment, the LI was lower when compared to the PBS- counterpart at 2 h (*P*<0.05), indicating that the stimulatory effect of fasting on p27 phosphorylation was diminished. In addition, TGFβ1 was still active as immunostaining remained lower than the respective control group (*P*<0.05).

### TGFβ1 and p27 regulators

Because we detected that TGFβ1 increased p27 levels and decreased its phosphorylation in the gastric mucosa, we also investigated whether cell-cycle related proteins involved in p27 stability would also be influenced by fasting and TGFβ1 gavage.

**Figure 2:**
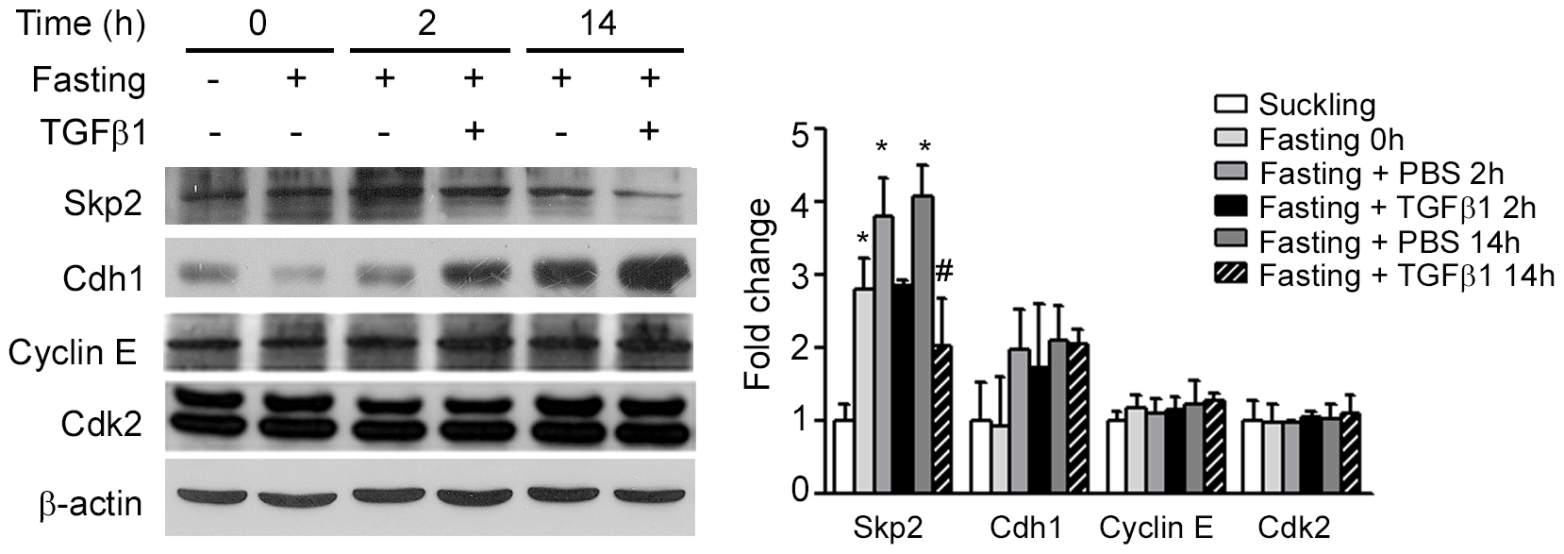
TGFβ1 effects on p27 regulators in the gastric mucosa of 14-day-old rats during fasting. Representative immunoblotting for Skp2, Cdh1, Cyclin E and Cdk2 proteins in the gastric mucosa of suckling and fasted pups after 0, 2 and 14β1 gavage. β-actin was used as loading control. Each band represents one animal and fold changes are shown as means ± SD for each protein. (*n*) = 3–4 pups/group. Analyses were conducted in blots ran in triplicates. * *P*<0.05 *vs* suckling group. ^#^ *P*<0.05 *vs.* respective PBS-treated group.

We found that Cyclin E, Cdk2 and Cdh1were not significantly affected by treatment (Fig. 2). However, we observed Skp2 augment after the onset of fasting when compared to suckling control group (*P*<0.05). TGFβ1 significantly reduced Skp2 after 14 h of administration (*P*<0.05).

### Specific role of TGFβ1 on p27 levels

**Figure 3:**
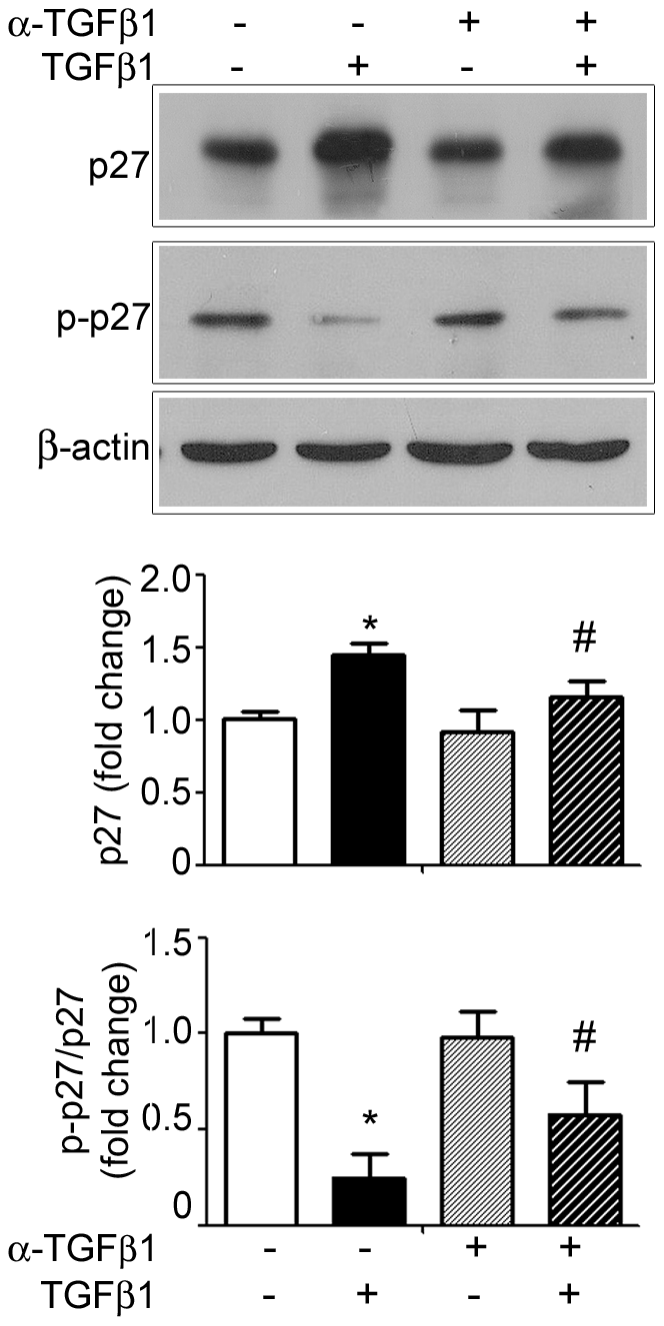
TGFβ1 direct effects on p27 and phospho-p27^Thr187^ in the gastric mucosa of 14-day-old rats during fasting. Representative immunoblotting for p27 and phospho-p27^Thr187^ in the gastric mucosa of pups fasted and gavaged with PBS, TGFβ1, α-TGFβ1 or TGFβ1+α-TGFβ1. All samples were collected after 2 h of treatment. β-actin was used as loading control. Each band represents one animal and fold changes are shown as means ± SD for p27 levels and for the ratio of p-p27/p27. (*n*) = 3 pups/group. * *P*<0.05 when compared to fasted-control group, ^#^ *P*<0.05 *vs.* respective counterpart. Analyses were conducted in blots ran in triplicates.

In order to evaluate the specific effect of exogenous TGFβ1 on p27 concentration and phosphorylation, we used an antibody against the peptide to neutralize its activity. We observed that TGFβ1 gavage increased p27 levels when compared to the group treated with PBS (*P*<0.05) (Fig. 3), as expected and shown above. When α-TGFβ1 was administrated together with the peptide (α-TGFβ1+TGFβ1) during fasting, p27 concentration decreased regarding TGFβ1 counterpart (*P*<0.05), and it was restored to the control levels (PBS- group), indicating the neutralization of TGFβ1 effects (Fig. 3).

Additionally, we compared the direct role of TGFβ1 on phosphorylation control, and after confirming that TGFβ1 gavage reduced phospho-p27^Thr187^ levels (*P*<0.05) (Fig. 3), we verified that the combined use of α-TGFβ1+TGFβ1 significantly increased phospho-p27^Thr187^/p27 ratio when TGFβ1- treated group was considered (*P*<0.05) (Fig. 3). Of note, for both total and phosphorylated proteins, the administration of α-TGFβ1 during fasting did not exert any response (Fig. 3)

### TGFβ1 role on p27 expression and protein synthesis

**Figure 4:**
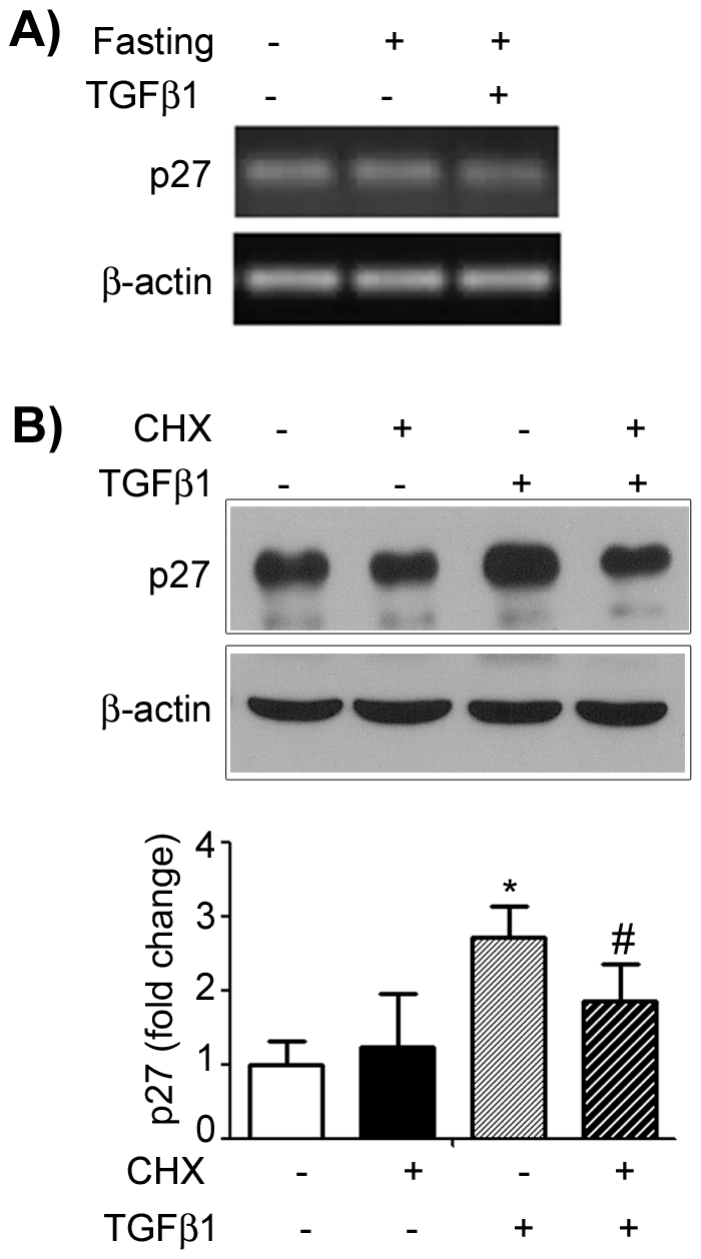
TGFβ1 effects on p27 expression and synthesis in the gastric mucosa of 14-day-old rats during fasting. (A) *p27* mRNA levels in the gastric mucosa of pups after 2 h of PBS and TGFβ1 gavage. *β-actin* was used as control. (*n*) = 5 pups at each condition. Analyses were performed in duplicates. (B) Immunoblotting for p27 protein levels in fasted pups treated as indicated with cycloheximide (CHX) to inhibit protein synthesis. All samples were collected after 2 h of treatment. β-actin was used as loading control. Each band represents one animal and fold changes are shown as means ± SD. (*n*) = 3 pups/group. * *P*<0.05 *vs.* fasted-control group; # P<0.05 *vs.* TGFβ1 group. Analyses were conducted in blots ran in triplicates.

In order to study *p27* expression in the gastric mucosa of suckling rats submitted to fasting and TGFβ1 treatment (2 h), we used RT-PCR to detect it. As shown in Figure 4A, we did not observe significant differences, and so we did not conduct qRT- PCR analyses.

Next, to evaluate if TGFβ1 might be involved in the control of p27 translation, we administered cycloheximide (CHX) to the pups to inhibit protein synthesis. We confirmed that TGFβ1 increased p27 concentration during fasting (*P*<0.05) (Fig. 4B), and we found that CHX (TGFβ1+CHX) reduced p27 levels when compared to the group treated with the peptide (*P*<0.05) (Fig. 4B).

### TGFβ1 role on p27 degradation

In order to understand how long it takes to the gastric mucosa to degrade p27 during fasting and after TGFβ1 gavage, we collected samples from pups treated for 2 h and prepared total protein lysates that were incubated for progressive periods in the presence of degradation buffer containing recombinant p27 (p27r) [34].

**Figure 5:**
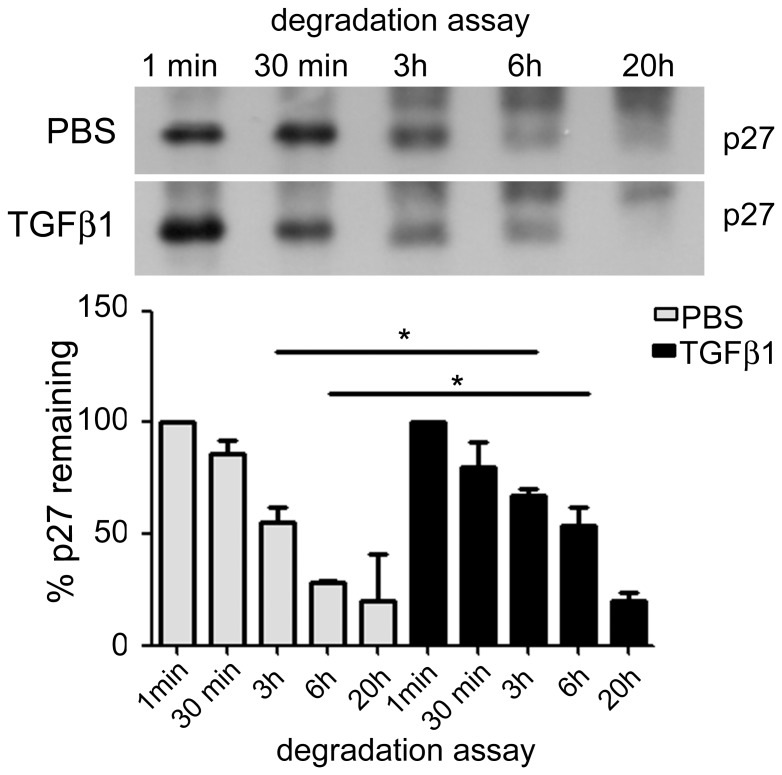
TGFβ1 reduces p27 protein degradation in the gastric mucosa of 14-day-old fasted rats. Immunoblotting for p27 during degradation assay, after 1, 30(p27r) protein. Samples were collected after 2 h of gavage with PBS or TGFβ1. The representative blots show the p27 band and a smear above it, which was derived from the degradation at each period of incubation. Each band represents one animal. Bars show the means ± SD of remaining p27r (%) based on relative density of each band over time compared with samples at 1 min. (*n*) = 4 pups at each condition. During the degradation assay, the % of remaining p27 decreased significantly in PBS- and TGFβ1- treated groups (*P*<0.05) (ANOVA). * *P*<0.05 for PBS *vs.* TGFβ1 treatment at 3 and 6 h of incubation. The assay was conducted in duplicates.

We observed that p27r was degraded in PBS- and TGFβ1- treated groups, as the decrease of p27 level was recorded for both conditions (*P*<0.05). However, higher concentrations of remaining p27r were detected in TGFβ1 samples when compared to the PBS counterparts after 3 and 6 h of degradation assay (*P*<0.05) (Fig. 5). Accordingly, we found that after 3 h of incubation, 69.6% of p27r were remaining in the lysates obtained from TGFβ1 group, whereas in PBS- gavaged pups, we recorded 55.5% from the initial p27r level (*P*<0.05). After 6 h, we detected 55.8% and 28% of remaining p27r, respectively in TGFβ1 and control pups (*P*<0.05) (Fig. 5).

## Discussion

In the current study, we showed for the first time that luminal TGFβ1 increased p27 levels in the rat gastric mucosa through the regulation of protein synthesis and degradation. This result was achieved when we used fasting condition as a physiological model to stimulate the proliferation of gland cells in rat pups [38], [39] and to challenge TGFβ1 response [6]. But also as important, we demonstrated that food restriction promoted p27 degradation. It is well established that reduced levels of p27 are directly related to tumor growth and poor prognosis in several types of carcinomas, especially due to the escape of cell cycle control. Herein, we showed that the non-transformed gastric mucosa responded to the physiological stimulus of fasting and to TGFβ1, allowing the study of the molecular mechanism involved in gastric growth.

During postnatal development, gastric p27 concentration is modulated in response to feeding condition, dietary pattern, growth factors and hormones, and the changes reflect on cell proliferation regulation [6], [46]–[48]. We observed that in suckling rats, whereas p27 levels were high, phosphorylation at Thr^187^ was low. However, fasting increased phospho-p27^Thr187^/p27 ratio and decreased the total concentration of the CKI. In addition, the combined record of nuclear phospho-p27^Thr187^ immunolabeling and the high distribution of positive cells along the gland (which represents the proliferative compartment during the first weeks of postnatal development) [38], [40] indicated that food restriction was directing p27 to degradation and consequently, it promoted cell proliferation as previously described [6], [49], [50]. Although previous reports have discussed the importance of p27 decrease during late G1 phase in multiple cell types submitted to different pro- proliferative stimuli in vitro [26], [31], [33], [51], [52], this is the first study to focus on the effects promoted by fasting condition on p27 phosphorylation and degradation in vivo.

During fasting, the components of meal are depleted from luminal content and the digestive metabolism is low, but interestingly, during suckling phase, food restriction also represents the lack of TGFβ and other milk-born growth factors. In addition, the gastric mucosa produces low levels of TGFβ1 and TGFβ3 [22]. De Andrade Sá et al. (2008) showed that the gavage of TGFβ1 during fasting inhibits gastric cell proliferation and induces apoptosis in the presence of high p27 levels [6]. We confirmed that TGFβ1 increased the concentration of p27, but we further observed that TGFβ1 reversed fasting- induced p27 phosphorylation, which might contribute to the low proliferative rates, previously described for this condition. Moreover, we demonstrated that these responses were specifically triggered by gavaged- TGFβ1 as indicated after the experiments with anti- TGFβ1. Accordingly, we showed that the neutralizing antibody was effective only when combined with the growth factor, suggesting that exogenous TGFβ1 had a direct role on the epithelium. Milk- born TGFβ was firstly studied in the 1990's [14], [15], when both the stability of the peptide and its effects were characterized. Of note, while rat pups remain suckling, growth factors from milk are available and active [16], [17], [53], and we currently showed that the exogenous TGFβ1 was able to specifically regulate p27 levels. Such results add important data to the understanding of TGFβ1 roles during gastric ontogenesis and cell cycle control.

Next, we investigated whether different molecules involved in the regulation of p27 levels and activity would be altered by fasting and TGFβ1 treatment. As Cyclin E/Cdk2 complex is part of the machinery required for p27 phosphorylation [27], [28], [49], we first checked for their concentration, and we observed that Cyclin E and Cdk2 were not influenced. This result corroborated previous studies that demonstrated that neither fasting nor early weaning changed the expression of these molecules [6], [46], [47]. However, because it was shown that gavage with TGFβ1 during fasting promoted a higher interaction between p27 and Cyclin E [6], we further evaluated other elements that might be involved in p27 degradation.

SCF E3 ligase complex is known to promote the degradation of cell cycle proteins and Skp2 is the nuclear adaptor responsible for the ubiquitylation of phospho-p27 and ultimately its degradation via proteasome [54]–[57]. The analysis of 98 patients with gastric carcinoma showed that tumors have a high expression of Skp2, which is inversely correlated to p27 levels [58], [59]. We detected that fasting induced a rapid increase in Skp2 concentration, and TGFβ1 might alter such response by decreasing Skp2 levels after 14 h of treatment. We also evaluated Cdh1 (APC/C^Cdh1^ complex targets Skp2) [60], [61], which promotes the accumulation of p27 [62], [63]. In colorectal carcinoma, concentrations of Cdh1 and p27 are very low when compared to healthy surrounding tissue, while the expression of Skp2 is inversely higher [64]. Our results were not significantly different, but they show a slight variation of this protein.

We checked for p27 expression and protein synthesis in the gastric mucosa, and found that only this latter process was modified by TGFβ1 gavage during fasting. These results are in agreement with other studies that suggest that p27 expression is not altered during treatment with TGFβ1 and indicate a post-transcriptional control [34], [50], [65]. If we consider the mechanisms that might be involved in the control of p27 translation, we can speculate about the role of miR- 221 and miR- 222. They control the p27-3′UTR region [66], repress the sensitivity to TGFβ and increase proliferation in breast cancer cells [66], [67]. Moreover, miR- 221 has been associated to a poor prognosis in gastric cancer [68].

Up to this point, we had demonstrated that TGFβ1 increased p27 levels by decreasing the phosphorylation at Thr^187^, and it did not affect expression, while synthesis was slightly altered. In 1995, Pagano et al. were able to monitor the degradation of p27, and they noted that proliferating cells broke this CKI more rapidly than the quiescent ones [26]. By using a similar assay [34] we observed that samples collected from the gastric mucosa of pups gavaged with TGFβ1 degraded a smaller amount of p27 at a slower rate when compared to fasted-control group. Such results suggested that TGFβ1 increased p27 stability, which might be directly involved in cell proliferation control in the gastric epithelium [6]. Conversely, fasting induced p27 cleavage, creating a proper condition for cell cycle progression. Similar data were described for endometrial glands at different physiological phases and in tumors [34].

Altogether, we demonstrated that the presence of TGFβ1 in the gastric lumen was able to increase p27 synthesis and also to reduce its phosphorylation at Thr^187^ and final degradation. We concluded that during gastric postnatal development, the regulation of p27 protein concentration represents one of the mechanisms used by rapidly proliferating cells to respond to milk-born TGFβ1 and food restriction. Hence, we suggest that the suckling period can be an important window of gastric growth to study the major apparatus involved in epithelial cycle control, which will dually govern both ontogenesis and tumorigenesis.
